# Trajectories of potentially inappropriate medication use among older adults in Saudi Arabia

**DOI:** 10.3389/fphar.2025.1568160

**Published:** 2025-04-29

**Authors:** Fouad F. Jabri, Yajun Liang, Kristina Johnell, Jette Möller

**Affiliations:** ^1^ Department of Biostatistics, Epidemiology and Public Health, College of Medicine, Alfaisal University, Riyadh, Saudi Arabia; ^2^ Department of Global Public Health, Karolinska Institutet, Stockholm, Sweden; ^3^ Department of Medical Epidemiology and Biostatistics, Karolinska Institutet, Stockholm, Sweden

**Keywords:** older adults, Beers criteria, potentially inappropriate medications, inappropriate prescribing, outpatients

## Abstract

**Background:**

The trajectories of potentially inappropriate medications (PIMs) among older adults have not been well studied. This study aims to determine the 3-year trajectories of PIM dispensation and their determinants in older adults in Saudi Arabia.

**Methods:**

A cohort study was carried out based on medical records from visits by 9,887 older adults (≥65 years) to outpatient clinics at King Saud University Medical City in Saudi Arabia from 2017 to 2019. PIMs were identified using the 2019 Beers Criteria, using the first category: medications that should be avoided by most older adults. Multinomial logistic regression was used to estimate the associations between clinical factors and the trajectories of PIM adjusting for sociodemographic factors.

**Results:**

The analysis showed that over 82% dispensed at least one PIM, with 55.9% having sustained PIMs, 17.9% having no PIMs, 14.0% starting PIMs, and 12.2% experiencing sporadic PIM dispensations. After adjustment, metabolic disorders (adjusted odds ratio [aOR]: 2.61, 95% confidence interval [95% CI]: 2.17–3.15), hypertensive diseases (aOR: 5.32, 95% CI: 4.67–6.07), diabetes mellitus (aOR: 10.22, 95% CI: 8.80–11.86), and diseases of the esophagus, stomach, or duodenum (aOR: 10.90, 95% CI: 7.39–16.09) were significantly associated with sustained PIM dispensation. With an increasing number of diagnoses we found an increasing odds for three trajectories (starting PIM (aOR range 1.56 to 5.82), sporadic PIM (aOR range 1.47 to 4.86), and sustained PIM (aOR range 3.91 to 37.3). Furthermore, an increasing number of medications was associated with higher odds for the same trajectories: starting PIM (aOR range 2.01 to 6.03), sporadic PIM (aOR range 1.50 to 7.10), and sustained PIM (aOR range 4.34 to 59.9).

**Conclusion:**

This study showed a high prevalence of sustained trajectories of PIMs over time. Further, several common diagnoses and a greater total number of medications were identified as being associated with different PIM trajectories.

## 1 Introduction

Medications provide therapeutic benefits—such as preventing disease onset, treating symptoms or complications, and curing diseases—but considerations of age-related changes should guide their use in older adults (Hutchison and Sleeper, 2015) as they can act differently in older and younger individuals due to physiological and pathological age-related changes, leading to different safety profiles and therapeutic outcomes (Fialová et al., 2018).

In 1991, Beers et al. (Beers et al., 1991) established the first explicit criteria for identifying potentially inappropriate medications (PIMs) for older adults aged 65 years or older. PIMs present more risks than benefits when prescribed to older adults due to their mechanisms of action and potential interactions with other substances, highlighting the need for safer therapeutic alternatives (Fialová et al., 2018; Beers et al., 1991; Laroche et al., 2007; Stock et al., 2014). PIM use has been linked to higher incidence of adverse drug events and hospitalizations (Xing et al., 2019), and therefore, increased healthcare costs, and negative health outcomes, including frailty, falls, and mortality (Hyttinen et al., 2019; Ma et al., 2023; Moon et al., 2024; Muhlack et al., 2017).

Despite guidelines aimed at reducing PIMs among older adults (By the 2023 American Geriatrics Society Beers Criteria^®^ Update Expert Panel, 2023; O'Mahony et al., 2023), their use remains high worldwide (Tian et al., 2023). Additionally, there has been a global upward trend in the use of PIMs over the past 2 decades (Tian et al., 2023). However, most research so far has concentrated on snapshot assessments of PIM use through cross-sectional designs.

Understanding the occasional and sustained use of PIMs in older adults is crucial for developing strategies to prevent exposure, which requires longitudinal data. To date, a few studies have offered valuable insights, particularly regarding sustained PIM use (Koyama et al., 2013; Roux et al., 2020; Canadian Institute for Health Information, 2018; Wang et al., 2019). For example, a study conducted in the United States found that the trajectories of PIM use among older adult women over a 10-year period did not follow a consistent pattern (Koyama et al., 2013).

Several factors associated with PIMs have been identified; these include diabetes mellitus, hypertension, comorbidity, medication count, and high frequency of healthcare facility visits (Chang et al., 2014; Nothelle et al., 2019; de Araújo et al., 2022; Jabri et al., 2023; Samara et al., 2023; Nigussie and Demeke, 2023). Factors associated with chronic PIM use include increased age, being male, and having a higher number of diseases and medications, diabetes mellitus and cardiovascular diseases (Roux et al., 2020). However, extensive research on the association between the duration of PIM use and the risk of adverse events, as well as various trajectories of PIM use and their associated risks, remains limited (Roux et al., 2020).

A recent study in Saudi Arabia has identified a high and increasing prevalence of PIMs among older adults (Jabri et al., 2023). However, to date, no studies have examined the within-individual trajectories of PIM use in Saudi Arabia and similar contexts in other Arab countries. These countries share cultural attitudes towards exchanging and sharing prescription medications (Alhomoud, 2020), patients’ misunderstandings of medication labels (Alburikan et al., 2018), and a healthcare system that provide the ability to dispense high-risk medications without a physician’s prescription (Alosaimi et al., 2016). Further, a study from Saudi Arabia found that many physicians were unaware of established lists of medications deemed inappropriate for older adults (Alharkan et al., 2024). Additionally, approximately half of them reported uncertainty or lack of confidence in prescribing for this vulnerable population (Alharkan et al., 2024), which is further concerning (Hutchison and Sleeper, 2015). Unfortunately, there is a significant gap in geriatric medicine training within both medical schools and postgraduate programs in Saudi Arabia (AlZamil et al., 2019). As a result, many junior physicians exhibit limited knowledge in geriatrics and PIMs, and there appears to be a lack of interest in pursuing careers in this important field (AlZamil et al., 2019; Al-Aama, 2016). These factors underscore the need to investigate PIM use trajectories among older adults in Saudi Arabia.

This study aims to determine the 3-year stability or change in PIM dispensations, and to identify the determinants associated with these trajectories among older adults in Saudi Arabia.

## 2 Materials and methods

### 2.1 Study design and setting

We conducted a cohort study using data from electronic health records of all outpatient clinics at King Saud University Medical City (KSUMC) in Riyadh, Saudi Arabia. This encompassed all outpatient clinics, including Cardiology, Endocrinology, Pulmonary, Orthopedics, Obstetrics and Gynecology, Dermatology, Urology, Family Medicine, Ophthalmology, and Neurology. KSUMC is a large multidisciplinary academic medical center with approximately 1,500 inpatient beds. It serves the public by providing comprehensive primary, secondary, and tertiary care, serving over one million outpatients annually.

### 2.2 Study population

**FIGURE 1:**
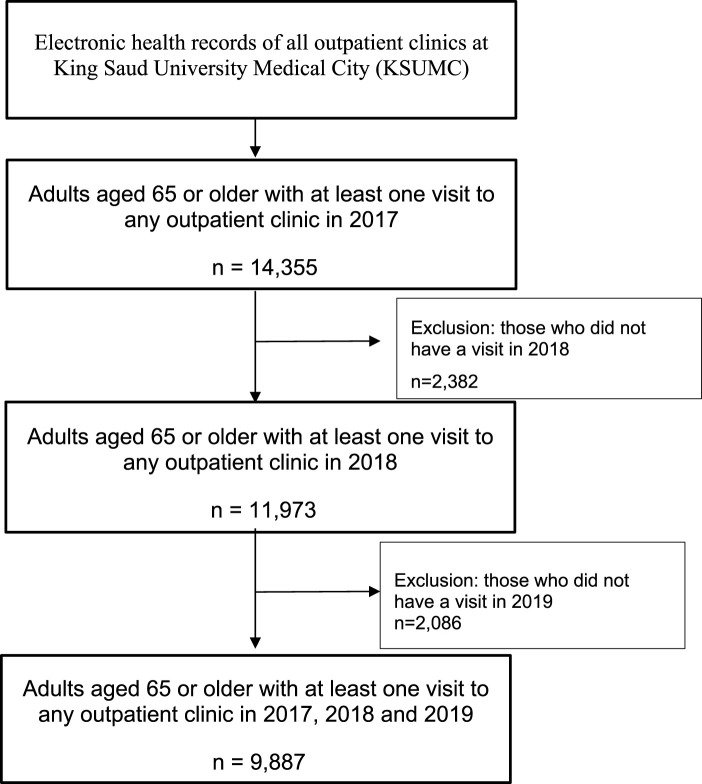
Flowchart of older adult selection for this study. Electronic health records of all outpatient clinics at King Saud University Medical City (KSUMC) Adults aged 65 or older with at least one visit to any outpatient clinic in 2017 n = 14,355 Exclusion: those who did not have a visit in 2018 n = 2,382 Adults aged 65 or older with at least one visit to any outpatient clinic in 2018 n = 11,973 Exclusion: those who did not have a visit in 2019 n = 2,086 Adults aged 65 or older with at least one visit to any outpatient clinic in 2017, 2018 and 2019 n = 9,887.

For this study, we used data from outpatients who were assessed for the first time in 2017, followed by re-evaluations in 2018 and again in 2019. At baseline, all individuals aged 65 or older who had at least one visit to the outpatient clinics at KSUMC were included. We excluded adults younger than 65 and those who were not followed up in 2018 or 2019. The final analysis included 9,887 older adults, as illustrated in Figure 1.

### 2.3 Data extraction

Data was retrieved from outpatient medical records using the Electronic System for Integrated Health Information (E-SiHi) at KSUMC in accordance with ethical guidelines. All visits to the outpatient clinics are recoded in the E-SiHI system. The information is recorded by physicians and pharmacists as part of their routine clinical practices. They were not informed or involved in this study’s implementation. The data was retrieved retrospectively after recording. The retrieved anonymized data included the following variables: date of visit, patients’ sex, nationality, age, and clinical characteristics, including diagnoses, dispensed medications (that is, medications that the patient has received from the pharmacy), and dispensation date.

### 2.4 The assessment of PIMs

The American Geriatrics Society (AGS) has compiled and published a range of PIMs in the 2019 Beers Criteria (By the 2019 American Geriatrics Society Beers Criteria^®^ Update Expert Panel, 2019). In this study, we employed the 2019 Beers Criteria to assess PIMs. Despite the availability of several tools for PIM identification, we opted for the Beers Criteria as they are more aligned with the characteristics of our dataset, which lacks extensive clinical information. The Beers Criteria’s non-disease-specific approach makes them particularly advantageous in contexts where clinical information is limited. Additionally, Beers Criteria have been validated in prior studies and demonstrate enhanced capability in detecting PIMs among community-dwelling older adults (Motter et al., 2018; Fajreldines et al., 2016). The 2019 Beers Criteria has five categories: 1) medications to avoid for most older adults, regardless of diagnosis or clinical conditions; 2) PIMs due to drug-disease interactions; 3) medications to be used with caution in older adults; 4) potentially clinically important drug-drug interactions; 5) medications requiring dosage adjustments based on kidney function. Due to the limited availability of clinical information and lab results in the medical records, our study specifically focused on the first category of the Beers Criteria to assess PIMs. The research team, which includes pharmacists, applied the 2019 Beers Criteria to the data by reviewing the dispensation records for each older adult from January 2017 to December 2019. Exposure to PIMs was identified if any dispensed medication was listed in the 2019 Beers Criteria from the AGS (By the 2019 American Geriatrics Society Beers Criteria^®^ Update Expert Panel, 2019).

### 2.5 Definitions of trajectories of PIMs

To analyze changes in PIM dispensation over time, older adults were categorized into four groups based on their 3-year PIM dispensation patterns. Older adults who had no PIM dispensations from 2017 to 2019 were considered as ‘No PIMs’ (NNN). Those receiving at least one PIM each year were labeled ‘sustained PIM dispensation’ (YYY). The group of older adults with no PIM dispensations in 2017 but started PIM dispensations in 2019 or in 2018 and continued in 2019 were classified as ‘starting PIM dispensation after 2017'(NNY, NYY). Lastly, those with PIMs in at least one but not all years were considered as ‘sporadic PIM dispensation’ (YNN, YYN, YNY, NYN). Switching between different PIMs was not considered a discontinuation of PIM dispensation, as the focus of this study is to evaluate the overall trajectories of PIM dispensation from 2017 to 2019.

### 2.6 Clinical variables

For each outpatient visit, the diagnoses were documented in the medical records (free text field with list of diagnoses such as depression, diabetes mellitus, hypertension). The diagnoses were then categorized into primary morbidity groups based on the blocks of the 22 chapters of the International Statistical Classification of Diseases and Related Health Problems 10th Revision (ICD-10)-WHO (WHO). A variable ‘number of diagnoses’ was created to reflect the total number of different diagnoses for each older adult based on the block levels of the ICD-10, and it was categorized into the following groups: 0, 1, 2 to 4, and 5 or more.

Dispensed medications were classified according to the Fifth Level of the Anatomical, Therapeutic, and Chemical (ATC) classification system (WHO Collaborating Centre for Drug Statistics Methodology). To determine the number of dispensed medications (excluding PIMs) within a 100-day period following the first dispensation date, we calculated the total number of medications dispensed within a 100-day period following the first dispensation date, in accordance with KSUMC’s medication refill guidelines, which recommend dispensing for 3 months at a time.

### 2.7 Covariates

Based on previous studies (Nothelle et al., 2019; Guaraldo et al., 2011; Hyttinen et al., 2017), the covariates include sociodemographic factors such as sex, age, and nationality, along with the number of visits to outpatient clinics. Sociodemographic information covering sex, nationality and age were assessed at baseline. The study population was divided into five age groups: 65–69 years, 70–74 years, 75–79 years, 80–84 years, and 85 years and older. Nationality was classified as either Saudi or non-Saudi. Number of visits to outpatient clinics at baseline (in 2017) were assessed based on date and time of visits, where each new visit code was counted as one. The sum of annual visits was categorized into the following groups: 1–2, 3–4, and 5 or more.

### 2.8 Statistical analysis

Descriptive analyses were conducted to describe the older adults with regard to sociodemographic characteristics, the prevalence of PIMs in 2017, and the most commonly dispensed PIMs. Multinomial logistic regression was employed to determine the association between clinical characteristics (diagnoses, number of diagnoses, and number of dispensed medications excluding PIMs) and trajectories of PIM dispensations. Two models were analyzed: model 1 was crude, and model 2 was adjusted for sociodemographic (sex, nationality, age groups) and number of visits to outpatient clinics. In the analyses, the reference group consisted of the older adults who did not receive any PIMs over the 3-year period, which is referred to as the NNN group. All variables analyzed in this study were complete, with no missing data. Statistical significance was set at p < 0.05. All statistical analyses were performed in the statistical software IBM^®^ SPSS^®^ Statistics (version 28).

### 2.9 Ethical approval

The study was approved by the Institutional Review Board of King Saud University under the Expedited Track Review (reference number 19/0470/RB; project number E-19–3808).

## 3 Results

The characteristics of the older adults are described in Table 1. In 2017, the mean age was 72.3 years. Approximately 95% of the older adults were Saudi, and 50.3% were male. The most prevalent diagnoses included diabetes mellitus, hypertensive diseases, and metabolic disorders. Approximately one-third of older adults had at least five different diagnoses over the whole 3-year period. More than 90% of older adults dispensed at least one medication in 2017 and continued to do so in the two subsequent years. The most frequently dispensed medications were atorvastatin, metformin, aspirin, pantoprazole, and cholecalciferol.

**TABLE 1:** Sociodemographic and clinical characteristics at baseline of older adults visiting outpatient clinics, (N = 9,887).

Characteristics	n	%
Sex		
Male	4,978	50.3
Female	4,909	49.7
Nationality		
Saudi	9,356	94.6
Non-Saudi	531	5.4
Age group (years)		
65–69	4,021	40.7
70–74	2,757	27.9
75–79	1,811	18.3
80–84	839	8.5
85+	459	4.6
Dispensed at least one medication	9,124	92.3
Number of diagnoses		
0	429	4.3
1	2,248	22.7
2–4	4,235	42.8
≥5	2,975	30.1
Number of dispensed PIMs		
0	3,501	35.4
1	2,718	27.5
2	1,998	20.2
3	1,057	10.7
4	417	4.2
5+	196	2.0

In 2017, a significant proportion of older adults (64.6%) received at least one PIM, with the most frequently dispensed PIMs including aspirin, pantoprazole, and meloxicam. Additionally, from 2017 to 2019, 82.1% of older adults were dispensed at least one PIM. Table 2 presents the prevalence of various PIM trajectories during this period.

**TABLE 2:** Trajectories of PIM dispensations and non-dispensation among older adults visiting outpatient clinics during 2017–2019 (n = 9,887).

Trajectories of PIMs	2017	2018	2019	n	%
Sustained PIM dispensations	Yes	Yes	Yes	5,527	55.9
No PIMs (no PIM dispensation)	No	No	No	1,765	17.9
Starting PIM dispensation after 2017 (without PIMs in 2017 but started PIM dispensations in 2019 or in 2018 and continued in 2019)	No	No	Yes	320	3.2
	No	Yes	Yes	1,068	10.8
Sporadic PIM dispensation	No	Yes	No	348	3.5
	Yes	No	No	232	2.3
	Yes	No	Yes	175	1.8
	Yes	Yes	No	452	4.6

A substantial majority, 73.8%, exhibited stability in their PIM dispensations and non-dispensation, as 55.9% had a sustained pattern of dispensing at least one PIM each year (YYY), and 17.9% did not dispense any PIMs (NNN). Conversely, 26.2% experienced changes in their PIM dispensation trajectories, with 14% initiating PIM dispensation after 2017 (Starting PIM) and 12.2% demonstrating sporadic PIM dispensation trajectories. Notably, of the 6,386 older adults who were dispensed at least one PIM in 2017, a substantial 86.5% continued to have PIM dispensations over the following 2 years. In contrast, only 3.6% had no recorded PIM dispensations during that same period.

Table 3 presents the prevalence of PIM trajectories stratified by demographic and clinical characteristics. The distribution of PIM trajectories among age groups, excluding sustained PIM dispensation, ranged from ∼15% to ∼20%. In contrast, the percentage of older adults with sustained PIM dispensation exceeded 50% across all age groups. The proportions of ‘starting PIM dispensation after 2017′and ‘sporadic PIM dispensation’ were comparable between males and females. Sustained PIM dispensations were prevalent among older adults for both females (58.5%) and males (53.3%).

**TABLE 3:** Distribution of PIM trajectories stratified by sociodemographic and clinical characteristics.

Characteristics	n	No PIMs (%)	Starting PIM dispensation after 2017 (%)	Sporadic PIM dispensation (%)	Sustained PIM dispensation (%)
Sex					
Male	4,978	20.2	14.4	12.1	53.3
Female	4,909	15.5	13.7	12.3	58.5
Nationality					
Saudi	9,356	18.3	14.0	12.0	55.7
Non-Saudi	531	10.4	15.1	15.1	59.5
Age groups					
65–69	4,021	17.7	14.3	12.8	55.2
70–74	2,757	19.3	14.6	10.9	55.2
75–79	1,811	16.6	13.0	12.8	57.7
80–84	839	17.0	14.3	11.9	56.7
85+	459	17.2	12.0	13.5	57.3
Number of visits to outpatient clinics					
1–2	4,717	24.1	19.5	11.7	44.6
3–4	2,843	14.1	9.6	11.9	64.5
5+	2,327	9.7	8.3	13.6	68.4
Number of diagnoses					
0	429	50.8	19.3	14.2	15.6
1	2,248	31.8	18.2	12.5	37.5
2–4	4,235	15.8	15.0	13.7	55.5
5+	2,975	5.5	8.7	9.6	76.1
Number of dispensed medications (excluding PIMs)					
0	890	57.5	18.5	13.6	10.3
1	965	35.2	20.3	13.2	31.3
2–4	3,530	19.0	17.5	13.1	50.5
5+	4,502	5.4	9.1	11.1	74.4
Diagnoses					
Hypertensive diseases					
No	5,127	27.7	14.8	13.9	43.5
Yes	4,760	7.2	13.2	10.4	69.2
Diabetes mellitus					
No	5,105	29.8	15.8	15.3	39.1
Yes	4,782	5.1	12.1	8.9	73.9
Metabolic disorders					
No	6,319	24.1	15.1	13.2	47.6
Yes	3,568	6.8	12.1	10.5	70.7
Disorders of eyelid, lacrimal system or orbit					
No	8,674	16.8	14.6	12.5	56.1
Yes	1,213	25.6	10.1	10.1	54.2
Diseases of esophagus, stomach or duodenum					
No	8,773	19.8	15.1	12.4	52.7
Yes	1,114	2.4	5.6	10.7	81.3

Older adults with fewer clinic visits, fewer diagnoses, and a lower number of medications represented a greater proportion of those with no PIMs or who started PIM dispensation after 2017. In contrast, sporadic PIM dispensation remained relatively stable regardless of the number of outpatient visits, diagnoses, or medications. Conversely, patients with a higher number of outpatient visits, more diagnoses, and an increased count of dispensed medications (excluding PIMs) constituted a greater proportion of those with sustained PIM dispensation. Moreover, older adults with metabolic disorders, diabetes mellitus, and diseases of the esophagus, stomach, or duodenum had higher prevalence of sustained PIM dispensation.

Table 4 shows the associations between clinical characteristics and PIM trajectories. Older adults with certain clinical characteristics were more likely to experience various PIM dispensation trajectories instead of a pattern without PIMs. The odds ratios (ORs) for diabetes mellitus, hypertensive diseases, metabolic disorders, and diseases of esophagus, stomach or duodenum, are generally high across different trajectories of PIMs. However, the ORs for sustained PIM dispensation are notably higher than those for older adults with changes in their PIM dispensation trajectories. This is further highlighted by the number of diagnoses and the number of dispensed medications (excluding PIMs).

**TABLE 4:** The association between clinical characteristics and PIM trajectories in comparison to the group without any dispensed PIMs, odds ratios (OR) with 95% confidence intervals (CI).

Characteristics		Starting PIM dispensation after 2017		Sporadic PIM dispensation		Sustained PIM dispensation	
		Crude	Adjusted	Crude	Adjusted	Crude	Adjusted
		OR (95% CI)	OR (95% CI)	OR (95% CI)	OR (95% CI)	OR (95% CI)	OR (95% CI)
Diagnosis							
Diabetes mellitus	No	Ref.	Ref.	Ref.	Ref.	Ref.	Ref.
	Yes	4.45 (3.75–5.29)	4.62 (3.88–5.49)	3.37 (2.82–4.03)	3.03 (2.52–3.65)	10.98 (9.5–12.71)	10.22 (8.80–11.86)
Hypertensive diseases	No	Ref.	Ref.	Ref.	Ref.	Ref.	Ref.
	Yes	3.40 (2.91–3.99)	3.48 (2.96–4.09)	2.85 (2.42–3.36)	2.57 (2.17–3.04)	6.10 (5.36–6.95)	5.32 (4.67–6.07)
Metabolic disorders	No	Ref.	Ref.	Ref.	Ref.	Ref.	Ref.
	Yes	2.85 (2.39–3.40)	2.94 (2.45–3.51)	5.31 (4.59–6.14)	4.65 (4.01–5.39)	2.84 (2.37–3.41)	2.61 (2.17–3.15)
Disorders of eyelid, lacrimal system or orbit	No	Ref.	Ref.	Ref.	Ref.	Ref.	Ref.
	Yes	0.45 (0.36–0.57)	0.45 (0.36–0.56)	0.53 (0.43–0.67)	0.45 (0.36–0.57)	0.63 (0.55–0.74)	0.45 (0.38–0.52)
Diseases of esophagus, stomach or duodenum	No	Ref.	Ref.	Ref.	Ref.	Ref.	Ref.
	Yes	3.01 (1.91–4.76)	3.01 (1.90–4.77)	7.04 (4.61–10.77)	6.60 (4.29–10.16)	12.62 (8.57–18.58)	10.90 (7.39–16.09)
Number of diagnoses	0	Ref.	Ref.	Ref.	Ref.	Ref.	Ref.
	1	1.51 (1.14–1.99)	1.56 (1.18–2.08)	1.41 (1.03–1.93)	1.47 (1.07–2.02)	3.85 (2.87–5.15)	3.91 (2.91–5.25)
	2–4	2.51 (1.90–3.30)	3.09 (2.33–4.11)	3.10 (2.28–4.20)	2.85 (2.08–3.91)	11.45 (8.60–15.26)	10.47 (7.81–14.04)
	≥5	4.12 (3.00–5.67)	5.82 (4.13–8.20)	6.20 (4.40–8.73)	4.86 (3.38–7.00)	44.67 (32.56–61.27)	37.30 (26.83–51.85)
Medications							
Number of dispensed medications (excluding PIMs) within a 100-day period following the first dispensation date	0	Ref.	Ref.	Ref.	Ref.	Ref.	Ref.
	1	1.79 (1.40–2.29)	2.01 (1.56–2.58)	1.58 (1.19–2.10)	1.50 (1.12–2.00)	4.94 (3.77–6.48)	4.34 (3.30–5.71)
	2–4	2.87 (2.33–3.53)	3.18 (2.57–3.93)	2.92 (2.32–3.67)	2.66 (2.10–3.37)	14.82 (11.67–18.83)	12.77 (10.02–16.27)
	≥5	5.20 (4.11–6.59)	6.03 (4.71–7.72)	8.64 (6.72–11.09)	7.10 (5.46–9.23)	76.43 (59.10–98.85)	59.90 (46.10–77.8)

For example, those with diabetes mellitus exhibited higher rates of starting, sporadic, and sustained PIM dispensations. After adjusting for sex, nationality, age, and number of outpatient visits, older adults with disorders of the eyelid, lacrimal system, or orbit were significantly less likely to have PIMs (adjusted odds ratio [aOR]: 0.45, 95% CI: 0.36–0.56). In contrast, metabolic disorders, hypertensive diseases, diabetes mellitus, and gastrointestinal diseases were all significantly associated with 3-year sustained PIM dispensation. Additionally, patients with five or more diagnoses showed an increase in the aOR for sustained PIM dispensation (aOR: 37.30, 95% CI: 26.83–51.85). Older adults with five or more dispensed medications had significantly higher aOR for sustained PIM dispensation, reaching as high as 59.90, 95% CI: 46.10–77.8.

## 4 Discussion

### 4.1 Main findings

This study shows distinct trajectories of PIM dispensations over time. We found that PIM dispensations are common, with approximately two-thirds of older adults receiving PIMs in 2017, and a substantial portion (55.9%) continuing to use PIMs over time. Our study indicates that both certain diagnoses and the total number of diagnoses and dispensed medications are strongly associated with the likelihood of various PIM dispensation patterns, particularly sustained PIM dispensation. Specifically, metabolic disorders, hypertensive diseases, diabetes mellitus, or gastrointestinal diseases increase the likelihood of different trajectory patterns of dispensed PIMs compared to having no PIMs.

Our findings are consistent with previous studies indicating that around two-thirds of older adults in outpatient settings are exposed to PIMs in Saudi Arabia (Alturki et al., 2020; Alharbi et al., 2022). This study also aligns with previous studies conducted in several developed countries, which reported a high prevalence of sustained PIM use over time among older adults across various healthcare settings (Canadian Institute for Health Information, 2018; Pineda et al., 2024; Schneider et al., 2019). For example, a study analyzing community pharmacy dispensing records revealed a high prevalence of sustained exposure to PIMs over a 3-year period among older patients with chronic diseases (Wang et al., 2019).

Our study shows that once PIMs are prescribed and dispensed, they are likely to be repeatedly dispensed over time, with only a small proportion of older adults discontinuing their use. Of the older adults who received PIMs in 2017, only 3.6% had no recorded dispensations over the next 2 years. A study analyzing population-level medication claims among older adults across all Canadian provinces revealed a decline in the prevalence of sustained PIM use, decreasing from 33.9% in 2011 to 31.1% in 2016 (Canadian Institute for Health Information, 2018). This modest change in proportion may be partly due to the challenges associated with modifying physician prescribing behaviors (Stock et al., 2014).

This study also highlights the association between the presence of multiple diseases, the use of multiple medications, and the risk of sustained PIM use. Consistent with our findings, Fernández, A., et al. (Fernández et al., 2021), reported that a greater number of diagnoses and a higher medication count were associated with sustained PIM dispensations in their 2-year follow-up study of community-dwelling older adults. Our results also align with those of Ble, A., et al. (Ble et al., 2015), which indicated that higher medication counts correlate with an increased risk of long-term PIM use. Furthermore, our findings reveal dose-response relationships between medication count and the likelihood of different PIM trajectories. Challenges to deprescribing among older adults contribute to the ongoing accumulation of medications over time (Woodford, 2024).

The association between specific diseases and the diverse patterns of PIM dispensation highlights significant differences. Older adults diagnosed with conditions affecting the eyelid, lacrimal system, or orbit were less likely to dispense PIMs. This may be attributed to the fact that many treatments for these conditions are topical rather than systemic, thereby decreasing the need for medications that could be deemed inappropriate for older adults (By the 2019 American Geriatrics Society Beers Criteria^®^ Update Expert Panel, 2019). In contrast, older adults with metabolic disorders or diseases of the esophagus, stomach, or duodenum showed a significant association with PIM dispensation across all types of trajectories. Additionally, our findings align with previous research by Roux, B., et al. (Roux et al., 2020), which identified hypertension and diabetes as significant predictors of sustained PIM dispensation. The chronicity of conditions and the high prevalence of comorbidities in older adults with diabetes and hypertension help elucidate these associations. Such disease complexities often necessitate multiple medications, thereby increasing the complexity of the medication regimen and the potential for PIMs.

In contrast to our findings, Ble, A., et al. (Ble et al., 2015), reported that patients with hypertension had a reduced risk of long-term PIM use. These differing associations may stem from variations in study populations, healthcare settings, and methodologies, including the use of a modified list of Beers criteria and the focus on only a subset of PIMs in the study by Ble, A., et al. (Ble et al., 2015). Furthermore, cardiovascular conditions within the UK Quality and Outcomes Framework benefit from enhanced monitoring, and certain high-risk medications in the UK carry explicit warnings about cautious prescribing for patients with risk of cardiovascular events (Ble et al., 2015). Currently in Saudi Arabia there is a lack of established guidelines addressing high-risk medications and their associated warnings for patients with cardiovascular vulnerabilities. Additionally, issues related to underreporting of prescribing data and the validity of chronic disease information in electronic clinical records may also contribute to the observed differences (Ble et al., 2015). Such discrepancy highlights the differing dynamics of PIM use across various populations and healthcare contexts, suggesting that contextual factors such as variations in prescribing practices and guidelines can significantly influence PIM use among older adults (Tian et al., 2023; Alhomoud, 2020; Alosaimi et al., 2016).

### 4.2 Strengths and limitations

The strengths of this study lie in its large sample size. Importantly, there was no recall bias since data was from medical records.

However, several limitations must also be acknowledged. As this is a hospital-based study, the results may be subject to selection bias since outpatients are different from community-dwelling older adults because these individuals are likely to have more health conditions and to being prescribed multiple medications, including PIMs. The findings may not be fully generalizable to the community-dwelling older adults.

Additionally, the diagnoses in our study are based on those recorded by physicians, and no validation studies have been conducted to assess their accuracy. As a result, there is a possibility of incorrect diagnoses or diagnostic errors, which could influence prescribed medications and potentially skew the evaluation of PIM use in our study. However, since we specifically focused on the first category of the Beers Criteria—medications to avoid for most older adults, regardless of diagnosis—it is unlikely that any incorrect diagnoses will significantly affect our evaluation of PIM use. Furthermore, KSUMC is a large, multidisciplinary academic medical center, and to our knowledge, the physicians are welltrained and competent. Therefore, if diagnostic errors do occur, they are likely to be random and minimal. We also believe that using medical records enhances the reliability of our data compared to self-reported data, which is more susceptible to information bias, including recall and memory bias.

Our study lacks information about over-the-counter (OTC) medications purchased from community pharmacies and some OTC medications can be classified as PIMs. Further some medications can be dispensed both as prescribed and without a prescription as OTC medications, such as aspirin and other non-steroidal anti-inflammatory drugs. The lack of information about OTC purchased PIMs may have therefore led to an underestimation of PIM use, although equally so across the 3 years under study. Moreover, we do not know the level of adherence, as we rely on dispensations, and this does not necessarily indicate that the medications were actually taken or used according to prescription. Nonetheless, some studies have reported high levels of adherence to PIMs among older adults (Yilmaz and Colak, 2018; Miyazaki et al., 2020).

Another limitation is that we treated baseline visits (in 2017) as a static variable rather than as a time-varying confounder. This approach may have led to biased estimates regarding the trajectory of PIMs, as the effect of clinic visits on the outcome could change over time. By not accounting for the variability in visit frequency throughout the study period, we may have inadequately adjusted for confounding factors, potentially underestimating the impact of visit patterns on the trajectory of PIMs.

### 4.3 Implications

The study highlights the need for improved prescribing practices and medication reviews to ensure the safety of older adults. In Saudi Arabia, the health of older adults and medication management are key priorities for the Ministry of Health (Alotaibi et al., 2022). However, the limited availability of medication review services in hospitals and pharmacies contributes to sustained PIM use, as these services have been shown to be effective in reducing prescriptions of PIMs (Spinewine et al., 2012; Alhaddad, 2019; Shadid et al., 2020; Alotaibi et al., 2023). Research demonstrates that pharmacist-led deprescribing interventions and medication reviews effectively discontinue PIMs (Ammerman et al., 2019). Promoting interdisciplinary collaboration between hospital physicians and community pharmacists can further enhance this effort (Ammerman et al., 2019; Chivapricha et al., 2021; Alcusky et al., 2021; Rodrigues et al., 2022; Zhou et al., 2023). Cultural attitudes towards prescription sharing (Alhomoud, 2020), misunderstandings of medication labels (Alburikan et al., 2018), and dispensing high-risk medications—such as psychotropic drugs, anticoagulants, insulins, and antiepileptics—without proper oversight (Alosaimi et al., 2016) may contribute to the prevalence of PIM use. Additionally, inadequate geriatric training for physicians undermines their confidence in prescribing, and limits their understanding of PIMs, leading to insufficient assessments of geriatric patients and their medication regimens, thereby increasing the likelihood of PIM prescriptions (Hutchison and Sleeper, 2015; Alharkan et al., 2024; AlZamil et al., 2019; Al-Aama, 2016; Voigt et al., 2016). Therefore, targeted educational initiatives and policy changes can be important to decrease the risks PIM pose to the health of older adults.

## 5 Conclusion

The dispensation of PIMs exhibited varying trajectories across a 3-year period among Saudi Arabian older adults. The use of multiple medications and the presence of multiple diseases were associated with the trajectory of PIM dispensation. Our findings underscore the need for recommendations on safer prescribing practices as well as initiatives to promote deprescribing protocols. To further validate these findings and identify further factors influencing PIM trajectories, future research should be conducted in community-based settings and include OTC medications.

## Data Availability

The raw data supporting the conclusions of this article will be made available by the authors, without undue reservation.
